# Clinical value of macrogenome next-generation sequencing on infections

**DOI:** 10.1515/biol-2022-0938

**Published:** 2024-09-09

**Authors:** Benfa Han, Xiaoli Zhang, Xiuxi Li, Mei Chen, Yanlin Ma, Yunxia Zhang, Song Huo

**Affiliations:** Department of Infectious Diseases, Southern Central Hospital of Yunnan Province, The First People’s Hospital of HongHe State, Honghe, 661000, Yunnan, China; Department of Pharmaceutical, Southern Central Hospital of Yunnan Province, The First People’s Hospital of HongHe State, Honghe, 661000, Yunnan, China

**Keywords:** intracranial infection, pathogen detection, mNGS technology, clinical analysis, efficacy evaluation

## Abstract

Intracranial infection (ICI) is a frequent and serious complication after neurosurgery. Macrogenome next-generation sequencing (mNGS) technology can provide reference for clinical diagnosis and treatment of ICI. This work aimed to explore the application value of mNGS technology in analyzing the clinical characteristics of human immunodeficiency virus (HIV) infection and ICI after neurosurgery. A total of 60 patients with ICI were enrolled as the research objects, all patients underwent routine cerebrospinal fluid analysis and traditional pathogen detection, followed by mNGS genome analysis. Using clinical diagnosis of ICI as the gold standard, the sensitivity, specificity, positive predictive value, and negative predictive value for both detection methods were calculated. Receiver operating characteristic curves were constructed to assess the area under the curve (AUC) for evaluating the clinical value of mNGS in suspected intracranial infectious pathogen diagnosis. Results showed a positivity rate of 71.67% (43 cases) with mNGS compared to 28.33% (17 cases) with traditional pathogen detection methods, demonstrating a significant difference (*P* < 0.05). The sensitivity of mNGS for detecting ICIs was 83.7%, significantly higher than the 34.88% observed with traditional methods (*P* < 0.05). The pathogen detection rate of mNGS was higher than traditional methods (*P* = 0.002), with an AUC of 0.856 (95% CI: 0.638–0.967), significantly greater than the AUC of 0.572 (95% CI: 0.350–0.792) for traditional methods (*P* < 0.05). mNGS successfully identified microorganisms such as *Cryptococcus*, *Propionibacterium*, *Staphylococcus*, *Corynebacterium*, *Micrococcus*, and *Candida* associated with ICIs. These findings underscore the clinical applicability of mNGS technology in analyzing the characteristics of HIV infection and ICI post-neurosurgical procedures. This technology enables more accurate diagnosis and treatment of ICIs, providing valuable insights for developing effective therapeutic strategies.

## Graphical abstract

**Figure:**
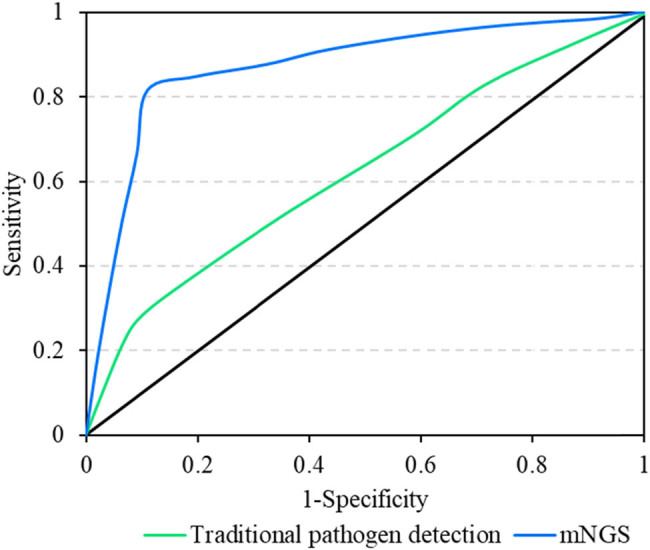

## 1 Introduction

Infectious disease is an important cause of death worldwide. There are many types of pathogens that infect the human body. Different environmental factors and irrational use of antibacterial drugs make the pathogens diverse and complicated [1]. Intracranial infection (ICI) is a common neurological disorder. Clinical features include headache, disturbance of consciousness, memory loss, movement disorder, and epilepsy [2]. Intracranial hypertension manifests as nausea, vomiting, and blurred vision; while intracranial inflammation manifests as fever, neck stiffness, and meningeal irritation [3]. Neuroimaging shows multiple intracranial lesions with different sizes. Common imaging findings include diffuse white matter lesions, brain parenchymal lesions, and intracranial space-occupying lesions. Some patients also show weight loss, fatigue, night sweats, lymph node enlargement, etc. [4,5]. The clinical manifestations of human immunodeficiency virus (HIV) infection with ICI may be similar to other diseases, so it is necessary to comprehensively consider the clinical symptoms, signs, laboratory tests, neuroimaging, and other information for diagnosis. The success of its treatment often depends on accurate diagnosis and timely treatment of pathogenic bacteria. However, routine examinations such as enhanced cranial MRI, blood culture, cerebrospinal fluid (CSF) culture, CSF routine, CSF cytology, CSF multiplex polymerase chain reaction (PCR), and CSF smear staining still fail to identify the pathogen in ICI patients, increasing the difficulty in treatment [6,7].

Macrogenome next-generation sequencing (mNGS) technology is a new type of deoxyribonucleic acid (DNA) sequencing technology, also known as second-generation sequencing technology [8]. It quickly and efficiently obtains a large amount of DNA sequence information by splitting complex DNA samples into millions of DNA fragments and sequencing these fragments simultaneously [9,10]. This technology has the characteristics of high throughput, high resolution, high sensitivity, and high specificity, and can obtain massive DNA sequence information in a relatively short period of time, thus providing a great opportunity for research in the fields of molecular biology and bioinformatics. Strong support [11,12]. In recent years, rapid advancements in mNGS technologies have significantly expanded their application in the analysis of microbial community structures and functions. Microbial communities, integral ecosystems within the human body, play crucial roles in human health and disease progression. Traditional first-generation sequencing technologies primarily focused on determining individual DNA sequences, thereby providing a solid foundation for genomic analysis [13,14]. However, these technologies have notable limitations when analyzing complex microbial communities [15,16]. In contrast, metagenomic NGS technologies efficiently and rapidly acquire extensive DNA sequence information from diverse microbial communities. Moreover, they deeply elucidate critical features such as complex metabolic pathways, mechanisms of resistance, and pathogenicity during infection processes [17]. mNGS can unbiasedly detect a variety of pathogenic microorganisms (including viruses, bacteria, fungi, and parasites) through DNA sequencing of clinical samples, and is gradually applied to the detection of clinical infectious disease pathogens. However, there are still confusions in clinical indications, experimental procedures, quality management, performance verification, and report interpretation of this technology [18]. In the traditional concept of microorganisms, the parts of the human body that do not have bacteria or other active microorganisms under normal physiological conditions are sterile parts [19]. During mNGS detection, low concentrations of microbial nucleic acids may be detected in normal sterile body fluids, but no living microorganisms exist. The diagnostic value of general sterile site specimens is better than that of normal bacterial site samples [20]. With the development of mNGS technology, metagenomic detection of CSF has been gradually introduced into the diagnosis of ICI. Compared with traditional examinations, metagenomic testing has the advantages of high sensitivity, high specificity, and high throughput. It can quickly identify pathogens in a shorter time and further guide doctors’ treatment decisions [21,22].

If ICI is not treated in time, it will endanger the life of the patient. Therefore, it is necessary to find the pathogen as soon as possible, make accurate diagnosis and treatment, avoid the abuse of infectious drugs, and improve the success rate of treatment. Therefore, this work conducted CSF metagenomic detection on HIV-infected patients with ICI and non-HIV-infected ICI patients, and combined with the original data of the metagenomic test and the treatment effect after adjustment, to understand the HIV-infected patients with ICI and their classification of ICI pathogens in non-HIV-infected patients. It was hoped to further improve the precise diagnosis of ICI, comprehensively analyze the application of metagenomic testing in ICI, provide more accurate and scientific methods for the diagnosis and treatment of ICI, and promote the application of CSF metagenomic testing technology in clinical practice.

## 2 Materials and methods

### 2.1 Research objects

A retrospective analysis was made of 60 ICI patients admitted to Southern Central Hospital of Yunnan Province, The First People’s Hospital of HongHe State from Southern Central Hospital of Yunnan Province, The First People’s Hospital of HongHe State, January 2020 to December 2022, Southern Central Hospital of Yunnan Province, The First People’s Hospital of HongHe State, including 38 males and 22 females. They were 43–68 years (51.3 ± 4.23 years old in average).The patients’ information was collected and recorded, including age, gender, underlying diseases, intracranial imaging results, antibiotic treatment, and prognosis. All the patients had signed the informed consent form according to the research requirements before data collection. All data were recorded in the customized research form. Furthermore, the patient’s vital signs were stable, and the examination had no obvious contraindications. Patients who fully met the inclusion criteria in this study accepted the test regulations and signed the informed consent for the experiment, and the study was approved by the Ethics Committee of Southern Central Hospital of Yunnan Province, The First People’s Hospital of HongHe State.

According to the *Expert Consensus on Diagnosis and Treatment of Central Nervous System Infections in Neurosurgery (2021 Edition)* [23], the diagnostic criteria for ICIs are as follows [1]: core body temperature ≥38 or <36°C, accompanied by decreased consciousness and mental status, along with symptoms of increased intracranial pressure such as headache, nausea, vomiting, papilledema, neck stiffness, neurological deficits, and evidence of purulent CSF drainage [2]. CSF characteristics include turbidity, yellowish or purulent appearance, white blood cell count >100 × 10^6^/L, neutrophil percentage >70%, elevated protein (>45 mg/dL), decreased glucose level (<2.2 mmol/L), or CSF glucose/blood glucose ratio <0.4 [3]. Positive CSF cultures or Gram staining with normal glucose, protein, and cell count levels in the absence of symptoms are considered contaminated.

The patients enrolled had to satisfy the following criteria: (1) HIV-infected patients and other ICI or suspected ICI patients; (2) patients without clear pathogen after menstrual blood culture, CSF culture, CSF smear staining, CSF multiplex PCR, CSF routine, CSF biochemical, and other routine detections; (3) patients with poor efficacy after anti-infection treatment; (4) patients with no other mental illness; (5) patients with complete case information and laboratory test results; and (6) it was necessary to further clarify the pathogen and adjust treatment, and find out the basis for diagnosis and treatment.

The patients had to be excluded if they had any of following items: (1) patients with contraindications such as lumbar puncture and bone marrow puncture, (2) patients with incomplete case data, (3) patients who could not obtain CSF, (4) patients with non-infectious diseases, (5) patients who did not agree to mNGS sequencing and refused to sign the informed consent, (6) patients with congenital diseases, and (7) patients who could not obtain specimens for inspection.


**Informed consent:** Informed consent has been obtained from all individuals included in this study.
**Ethical approval:** The research related to human use has been complied with all the relevant national regulations, institutional policies and in accordance with the tenets of the Helsinki Declaration, and has been approved by the Ethics Committee of Southern Central Hospital of Yunnan Province, The First People’s Hospital of HongHe State.

### 2.2 Macrogenome sequence process

Macrogenome sequencing is a high-throughput sequencing technology that is mainly used to analyze the composition, function, and interaction of microorganisms in complex biological systems such as environmental samples, microbial communities, and genomes. The macrogenome group sequencing process was as follows.

Initially, DNAs or RNAs were extracted from environmental samples or organisms and then performed with quality testing and purification. Next, the DNA/RNA fragments in the sample were amplified by PCR, and then added to a specific DNA/RNA adapter system to establish a library.

Secondly, the Illumina, PacBio, and other high-throughput sequencing platforms were employed to sequence the established library to generate original sequence data.

Next, preprocessing steps such as quality control, filtering, removing contaminated sequences, and removing low-quality sequences were performed on the original sequence data to obtain high-quality sequence data.

In the next step, the sequence data were spliced and assembled to form a long continuous sequence, and then the sequence was assigned to different taxa or representative sequences through database annotation or blast comparison analysis.

After that, sequence data classification, species diversity analysis, community structure analysis, and functional annotation are analyzed by comparing sequence databases, cluster analysis, and functional annotation.

Finally, the analysis data were visualized. The results were displayed with various charts and network diagrams, and explained to yield reference for subsequent further research.

### 2.3 CSF smear staining

Specimens of CSF were obtained using the following specific steps. A puncture was made at the patient’s lumbar site and the CSF was withdrawn with a sterile syringe into a sterile centrifuge tube. First, a smear was taken, a drop of CSF specimen was dropped on a glass slide, and then quickly spread into thin slices with another slide glass to let it dry. Then, the smear was fixed, the prepared glass slide was immersed in 95% ethanol, and the cells were fixed to remove the water in the CSF. Next, the fixed slide was immersed in one or more dye solutions, which could be Graham staining, partial alkaline staining, acidic staining, etc. The staining time and the concentration of the staining agent varied according to the staining method and the substance to be stained. The slides were rinsed with deionized water or alcohol, and allowed to dry naturally in the air or with warm air. Finally, the stained glass slide was put under the microscope to observe the cell type, quantity, shape, and structure. Finally, the results were recorded.

### 2.4 Methods for statistics

Experimental data were processed with SPSS 26.0. *P* < 0.05 was considered as a significant difference. The count data were represented by rate (*n*, %). If the sample size was greater than 40 and the theoretical frequency of four grids was not less than 5, the chi-square test of the four-grid table was employed. When the sample size was greater than 40 but 1 ≤ theoretical frequency < 5, the correction formula was applicable. When the sample size was less than 40 or the theoretical frequency was <1, the probability was calculated using the exact probability method. The normality test and variance test were carried out on the measurement data, and the data subject to normal distribution and homogeneity of variance were expressed as mean ± standard deviation ( \[\overline{x}\pm s]\] ).

## 3 Results

### 3.1 Comparison of positive rates

**Figure 1:**
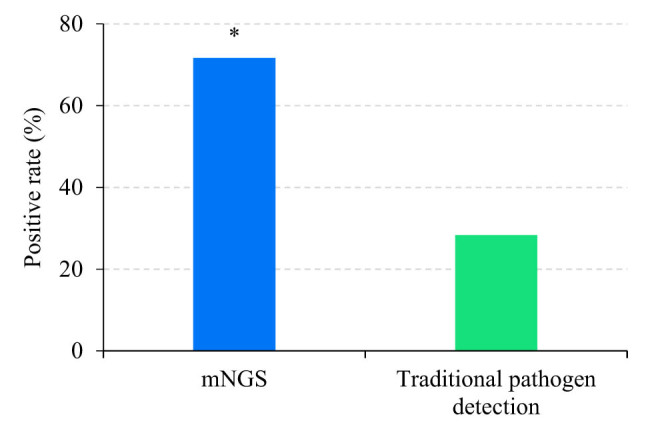
Comparison of positive rates. Note: * suggested a great difference with *P* < 0.05.

In a cohort of 60 patients, mNGS detected positive results in 43 cases, yielding a positivity rate of 71.67%. In contrast, traditional pathogen detection methods identified 17 patients with positive results, resulting in a positivity rate of 28.33%. The chi-square test revealed that mNGS had a higher positive rate of detecting pathogenic microorganisms, with *P* = 0.000 < 0.05 (Figure 1).

### 3.2 Diagnostic performance comparison

**Figure 2:**
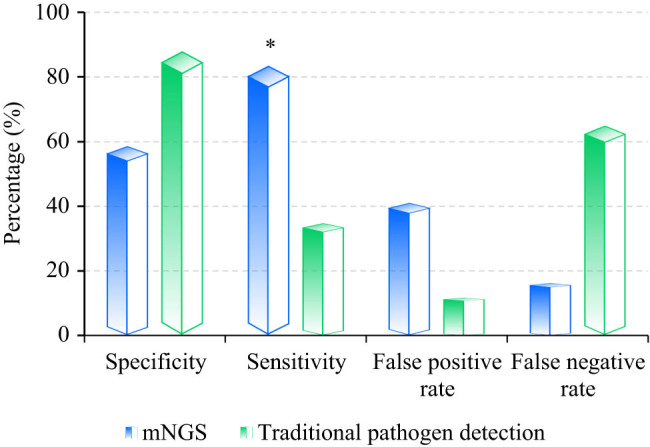
Diagnostic value of different detection methods. * suggested the great difference with *P* < 0.05.

Among 60 patients, mNGS yielded concordant results with ICI diagnosis in 36 cases, discordant results in 7 cases, true negative results in 17 cases, including 7 cases discordant with clinical diagnosis, and 10 cases concordant with clinical diagnosis. mNGS demonstrated an overall specificity of 58.8%, sensitivity of 83.7%, false positive rate of 41.2%, and false negative rate of 16.3% for ICI diagnosis. In comparison, traditional pathogen detection methods identified 17 patients with positive results, with 15 cases concordant with ICI diagnosis, and 43 patients with negative results, including 28 cases discordant with clinical diagnosis and 15 cases concordant with clinical diagnosis. Traditional methods exhibited an overall specificity of 88.24%, sensitivity of 34.88%, false positive rate of 11.76%, and false negative rate of 65.12% for ICI diagnosis. The specificity of mNGS was lower than that of traditional pathogen detection methods (*P* > 0.05), while its sensitivity was higher (*P* < 0.05) (Figure 2).

### 3.3 Different pathogen identification efficacy results

**Figure 3:**
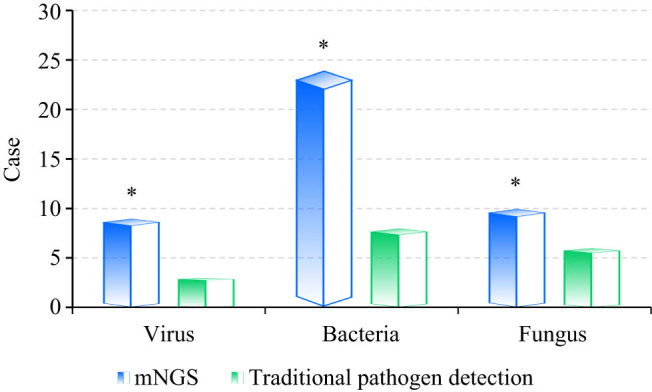
Different pathogen identification efficacy results. Note: * suggested a great difference with *P* < 0.05.

Figure 3 compared the differences in pathogen identification efficacy of two detection methods. As it demonstrated, nine cases of virus, 24 cases of bacteria, and 10 cases of fungi were detected using mNGS. In contrast, there were three cases of virus, eight cases of bacteria, and six cases of fungus in traditional pathogen detection. The chi-square test told that the detection of pathogenic microorganisms by mNGS was greatly higher based on that by traditional methods, showing a great difference with *P* = 0.002 < 0.05.

**Figure 4:**
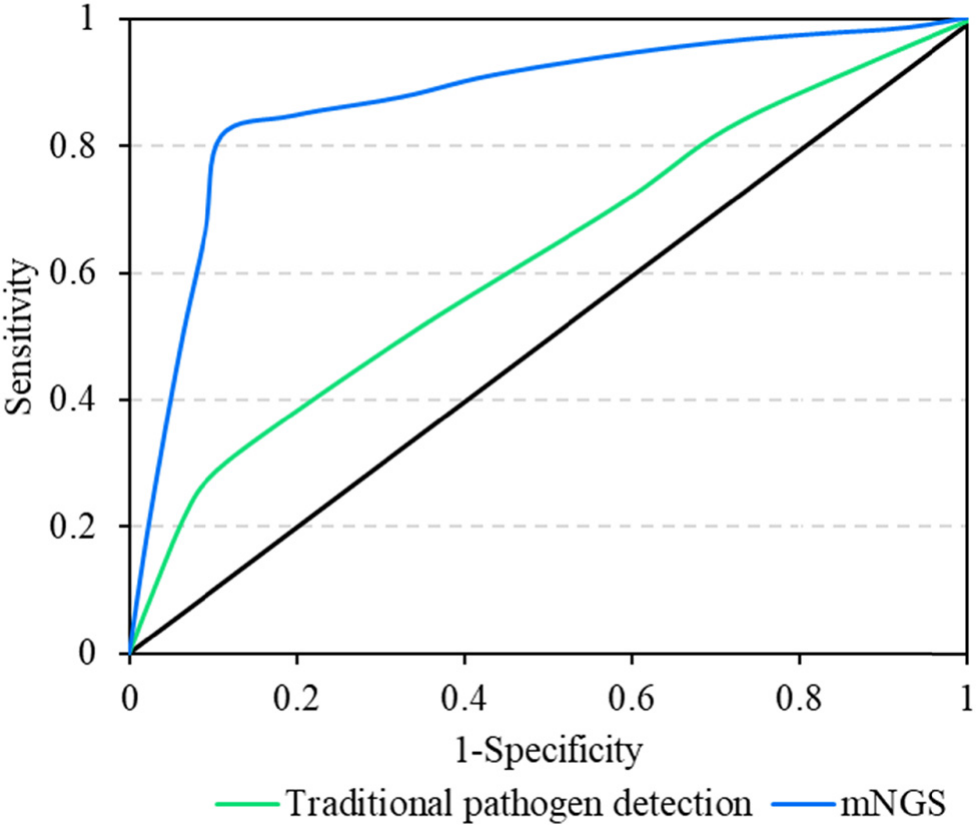
ROC curve.

The receiver operating characteristic (ROC) curve analysis revealed that the area under the curve (AUC) for mNGS was 0.856 (95% CI: 0.638–0.967), whereas for traditional pathogen detection methods, the AUC was 0.572 (95% CI: 0.350–0.792). The AUC of mNGS was significantly higher than that of traditional pathogen detection methods (*P* < 0.05) (Figure 4).

### 3.4 Cryptococcus

The results of the DNA process test (Table 1) were suspected to be positive. Bacteria, fungi, viruses, parasites, special pathogens (mycobacteria, mycoplasma/chlamydia), drug resistance genes, and virulence genes were not detected. Cryptococcal meningitis, AIDS, CSF, and blood cultures were all found to be cryptococcal. Laboratory tests were in line with clinical practice, and the patient was cured.

**Table 1:** Fungal cryptococcus information

Genus			Species	
Genus name	Relative abundance (%)	Number of sequences	Species name	Number of sequences
*Cryptococcus*	—	9	*Cryptococcus neoformans*	−7

### 3.5 Test results of suspected human microecological flora

As listed in Table 2, the suspected human microecological flora included *Propionibacterium, Staphylococcus, Corynebacterium, Micrococcus*, and *Candida*.

**Table 2:** Test results of suspected human microecological flora

Genus				Species	
Types	Genus name	Relative abundance (%)	Number of sequences	Species name	Number of sequences
G+	*Cutibacterium*	17.63	16,649	*C. neoformans*	−7
G+	*Staphylococcus*	8.34	7,880		
G+	*Staphylococcus*	8.34	7,880		
G+	*Corynebacterium*	5.67	5,355		
G+	*Micrococcus*	2.28	2,155		
Fun	*Candida*	0.01	17		

### 3.6 Test results for viral encephalitis

Table 3 lists the suspected human microecological flora in viral encephalitis. The DNA test result was suspected to be positive, and no bacteria, fungi, viruses, parasites, special pathogens (mycobacteria, mycoplasma/chlamydia), drug resistance genes, and virulence genes were detected. It was suspected as subacute hematogenous disseminated pulmonary tuberculosis or possible tuberculous meningoencephalitis. The CSF did not detect the pathogen, and the condition was improved after tuberculosis treatment.

**Table 3:** Suspected human microecological flora in viral encephalitis

Genus				Species	
Types	Genus name	Relative abundance (%)	Number of sequences	Species name	Number of sequences
G+	*Cutibacterium*	4.10	385	*Cutibacterium acnes*	286
G+	*Staphylococcus*	2.69	252	*Staphylococcus hominis*	102
G+	*Staphylococcus*	2.69	252	*Staphylococcus epidermidis*	56
G+	*Staphylococcus*	2.69	252	*Staphylococcus warneri*	26
G+	*Staphylococcus*	2.69	252	*Staphylococcus capitis*	24
G+	*Micrococcus*	0.56	53	*Micrococcus luteus*	49
G+	*Corynebacterium*	0.55	52	*Corynebacterium jeikeium*	5

### 3.7 Test results for the herpes simplex virus

It was suspected to be viral encephalitis, and herpes simplex virus type 1 was detected, which was consistent with the clinical situation, and the disease improved according to the treatment of the diseased encephalitis. Bacteria, fungi, RNA viruses, parasites, special pathogens (mycobacteria, mycoplasma/chlamydia), drug resistance genes, and virulence genes were not detected. The detected DNA viruses included human herpesvirus type 1 with a sequence number of 77, and human herpesvirus type 5 (CMV) with a sequence number of 4. The suspected human microecological flora of herpes simplex virus is shown in Table 4.

**Table 4:** Suspected human microecological flora in herpes simplex virus

Genus				Species	
Types	Genus name	Relative abundance (%)	Number of sequences	Species name	Number of sequences
G+	*Cutibacterium*	2.15	15,190	*C. acnes*	12,359
G+	*Staphylococcus*	1.18	8,348	*S. capitis*	2,357
G+	*Staphylococcus*	1.18	8,348	*S. epidermidis*	1,211
G+	*Staphylococcus*	1.18	8,348	*S. warneri*	696
G+	*Corynebacterium*	0.93	6,603	*S. capitis*	419
G+	*Micrococcus*	0.50	3,555	*M. luteus*	2,999
G+	*Moraxella*	0.33	2,330	*C. jeikeium*	2,252

### 3.8 Test results for viral encephalitis

The DNA test results were suspected to be negative, and no bacteria, fungi, viruses, parasites, special pathogens (mycobacteria, mycoplasma/chlamydia), drug resistance genes, and virulence genes were detected, as listed in Table 5. It was suspected as subacute hematogenous disseminated pulmonary tuberculosis or possible tuberculous meningoencephalitis. In the CSF, the pathogen was not detected, and the disease improved after the tuberculosis treatment.

**Table 5:** Suspected human microecological flora in viral encephalitis

Genus				Species	
Types	Genus name	Relative abundance (%)	Number of sequences	Species name	Number of sequences
G−	*Moraxella*	8.34	1,996	*Moraxella osloensis*	1,963
G+	*Cutibacterium*	2.58	617	*C. acnes*	489
G+	*Staphylococcus*	1.37	328	*S. hominis*	85
G+	*Staphylococcus*	1.37	328	*S. warneri*	71
G+	*Staphylococcus*	1.37	328	*Staphylococcus haemolyticus*	43
G+	*Staphylococcus*	1.37	328	*S. epidermidis*	39
G+	*Staphylococcus*	1.37	328	*Staphylococcus cohnii*	19
G+	*Staphylococcus*	1.37	328	*S. capitis*	18
G+	*Corynebacterium*	0.97	233	*Corynebacterium pseudogenitalium*	19
G+	*Micrococcus*	0.78	187	*M. luteus*	175

### 3.9 Test results for cryptococcal meningitis

The DNA test result was suspected to be positive, and no bacteria, parasites, drug resistance genes, and virulence genes were detected (Table 6). The fungus checked was *C. neoformans* with a sequence number of 14602018, and common colonizing bacteria in humans were detected. The detected viruses include human herpes virus 6A with sequence number 18, human herpes virus type 4 (EBV) with sequence number 19, and CMV with sequence number 1. Combined with the Mycobacterium complex, the sequence number was 2. Only for anti-fungal treatment improved, no anti-tuberculosis drugs were applied, and the disease was better.

**Table 6:** Test results for cryptococcal meningitis

Genus				Species	
Types	Genus name	Relative abundance (%)	Number of sequences	Species name	Number of sequences
G+	*Cutibacterium*	23.50	20,219	*C. acnes*	14,071
G+	*Staphylococcus*	12.32	10,596	*S. warneri*	6,597
G+	*Staphylococcus*	12.32	10,596	*S. epidermidis*	1,496
G+	*Corynebacterium*	3.02	2,603	*Corynebacterium kroppenstedtii*	391

### 3.10 Test results for conjunctivitis and ankylosing spondylitis

Table 7 displays the test results for conjunctivitis and ankylosing spondylitis. The DNA test result was suspected to be positive, and the bacteria were detected to be *Acinetobacter baumannii* with a sequence number of 193. The detected DNA viruses included human herpes virus type 7 with a sequence number of 1, and EVB with a sequence number of 1. Parasites, drug resistance genes, and virulence genes were not detected. Anti-tuberculosis treatment was effective.

**Table 7:** Test results for conjunctivitis and ankylosing spondylitis

Genus				Species	
Types	Genus name	Relative abundance (%)	Number of sequences	Species name	Number of sequences
G+	*Staphylococcus*	3.28	1,015	*S. warneri*	236
G+	*Staphylococcus*	3.28	1,015	*S. haemolyticus*	164
G+	*Staphylococcus*	3.28	1,015	*S. hominis*	134
G+	*Staphylococcus*	3.28	1,015	*S. capitis*	121
G+	*Staphylococcus*	3.28	1,015	*S. cohnii*	106
G+	*Staphylococcus*	3.28	1,015	*S. epidermidis*	73
G+	*Staphylococcus*	3.28	1,015	*Staphylococcus arlettae*	65
G+	*Cutibacterium*	1.54	479	*C. acnes*	381
G+	*Micrococcus*	0.74	229	*M. luteus*	211
G-	*Moraxella*	0.69	214	*M. osloensis*	208
G+	*Corynebacterium*	0.56	173	*M. luteus*	175

## 4 Discussion

There is a huge gap between routine detection technology and mNGS in the detection of infectious diseases. In severe central nervous system infection, bloodstream infection, and other diseases, the pathogen poses a great threat to the body, and if the treatment is not timely, severe septic shock will occur [24,25]. mNGS is suitable for the study of microbial communities in complex environments, including microbiomes, environmental samples, human microbiomes, etc. By sequencing these microbial communities, phase detection techniques and mNGS-related information such as genome information, population structure, metabolic pathway, and functional characteristics can be obtained, providing important data support for in-depth understanding of ecology, evolution, metabolism, and other aspects of microbial communities [26]. At the same time, mNGS technology can also be applied to research in the fields of genomic variation, expression profiling, single-cell sequencing, and human genetic diseases. In this work, CSF routine detection and CSF macrogenome detection were combined to improve the diagnostic accuracy of ICI. The positive rate of pathogens detected by mNGS was 71.67%, and that of traditional pathogen detection was 28.33%. mNGS demonstrated a sensitivity of 83.7% for ICI detection, surpassing the 34.88% sensitivity observed with traditional pathogen detection methods. Furthermore, mNGS exhibited a higher pathogen detection rate compared to traditional methods. The AUC for mNGS was 0.856 (95% CI: 0.638–0.967), significantly greater than the AUC of 0.572 (95% CI: 0.350–0.792) for traditional pathogen detection methods. These findings underscore mNGS’s effectiveness in capturing intracranial infectious pathogens and its superior accuracy and reliability in diagnosing ICIs. The advantages of mNGS technology in ICI diagnosis are evident, highlighted by its high sensitivity, accuracy, and capability to identify a diverse range of microbes. This capability provides clinicians with essential diagnostic insights, facilitating faster and more accurate determination of the etiology of ICIs. Moreover, it supports the development of personalized treatment strategies based on precise microbiological information. This work revealed that mNGS has a high diagnostic performance, which is consistent with the findings of Dellinger [27]. Weiss et al. [28] used mNGS for the detection of pathogenic bacteria in patients with pneumonia, mNGS for comprehensive early detection of *C. psittaci* pneumonia in patients with respiratory tract infections. Simultaneously early identification of co-infection will further improve the clinical management of these patients. Ma et al. [29] indicated that the next-generation sequencing of macrogenome showed a strain in the oral microbiota, and that the next-generation sequencing of macrogenome could help to determine the best treatment strategy for brain abscesses caused by oral pathogens. Macrogenome group next-generation sequencing can identify infectious agents in a target-independent manner. Hu et al. [30] indicated that mNGS may be a useful diagnostic tool for *T. gondii* encephalitis. The presence of *T. gondii* genome in CSF was further confirmed by *T. gondii*-specific PCR and Sanger sequencing. Since mNGS is capable of identifying all pathogens in a single run, it may be a promising strategy for exploring clinically causative pathogens of CNS infections with atypical features.

mNGS successfully identified various microbes associated with ICIs, including *Cryptococcus*, *Propionibacterium*, *Staphylococcus*, *Corynebacterium*, *Micrococcus*, and *Candida*. *Cryptococcus*, a common fungus, particularly causes severe ICIs in immunocompromised individuals, with HIV-infected patients being most vulnerable. It is widely distributed in the environment and primarily enters the human body through the respiratory tract, where its severity correlates closely with its affinity for the central nervous system [31,32]. *Propionibacterium*, commonly found on the skin and mucous membranes, can invade the intracranial space following surgery or trauma, contributing to intracranial abscess formation [33]. *Staphylococcus*, including *Staphylococcus aureus*, is a frequent pathogen in post-surgical ICIs, particularly associated with surgical wound infections [34]. *Corynebacterium* can lead to ICIs, especially in immunocompromised individuals or postoperative settings, causing various community-acquired and nosocomial infections such as bloodstream infections, catheter-related infections, endocarditis, septicemia, meningitis, pneumonia, skin and soft tissue infections, prosthetic joint infections, pyelonephritis, and liver abscesses [35]. *Candida*, a common fungal pathogen, particularly causes ICIs in patients on long-term antibiotics or immunosuppressive therapy. The successful identification of these microbes by mNGS highlights its high sensitivity in comprehensive detection and identification of potential pathogens associated with ICIs.

Through CSF smear staining, it is possible to understand the cells quickly and intuitively in CSF, and help doctors make a preliminary judgment on the disease. The mNGS technology enables high-throughput and high-sensitivity detection of ICI pathogens, and can detect multiple pathogens simultaneously. In this work, the mNGS sequencing technology detected a variety of ICI pathogens, including bacteria, fungi, and viruses, and some of the detection results were inconsistent with traditional detection methods, indicating that mNGS technology has great potential in the detection of ICI pathogens. Tu et al. [36] analyzed a rare case of varicella-zoster virus meningoencephalitis with meningoencephalitis suspected central nervous system leukemia, based on further research by mNGS technology, the final diagnosis was varicella-zoster disease meningoencephalitis with meningoencephalitis. The patients were treated with acyclovir and foscarnet, and repeated CSF studies revealed normal cell counts and proteins. No abnormal cells were found. Repeated brain MRIs also showed marked resolution of previously abnormal meningeal enhancement. Fang et al. [37] indicated that mNGS may be a useful diagnostic tool for CNS varicella-zoster virus infection. Since mNGS can identify all pathogens directly from CNS samples in a single run, it is beneficial to enhance the ability to diagnose CNS infections in patients with CNS infections. This study analyzed HIV-infected patients with ICI and non-HIV-infected ICI patients in Honghe Prefecture, patients whose blood culture, CSF culture, CSF routine, CSF cytology, CSF smear staining, and other routine examinations have not yet identified the pathogen, and suspected ICI patients could not be clearly diagnosed. All were tested for CSF using mNGS. In this work, the cryptococcal meningitis test results, DNA test results were suspected to be positive, only anti-fungal treatment improved, no anti-tuberculosis drugs were used, and the patient improved. In addition, this work found that the types of pathogens in ICI were related to factors such as infection route and patient age. Therefore, when formulating the treatment plan for ICI, it is necessary to accurately identify the pathogen to take corresponding treatment measures. The results of this study suggest that the mNGS technology can provide more accurate and comprehensive information for the identification of pathogens in ICI, and provide technical support for the formulation of individualized treatment plans. Overall, the results of this work revealed that mNGS technology has great potential in the detection of ICI pathogens, which can provide clinicians with more accurate and comprehensive pathogen information, and help guide the formulation and adjustment of ICI treatment plans. However, there are still some problems and limitations in this technology, which need further research and improvement.

## 5 Conclusion

The positive rate of pathogens detected by mNGS was sharply higher based on that of traditional pathogen detection methods, showing an observable difference with *P* < 0.05. Routine detection on CSF of HIV-infected patients with ICI often showed abnormalities such as increased cell number and increased protein content. Brain imaging examination usually manifests as intracranial lesions, including cerebral edema, focal lesions, etc. mNGS can more accurately identify the causative agent of ICI and provide more options for the treatment of the disease, exhibiting a high application value in the clinical analysis of HIV infection and ICI. At present, mNGS still is subject to some limitations, such as insufficient sequencing depth and amplification preference of specific gene fragments. In the future, the accuracy, reliability, and applicability of the technology can be further improved to better support the clinical diagnosis and treatment of ICI. However, ICI involves complex pathophysiological mechanisms and clinical manifestations. In the future, it can explore its pathogenesis and clinical features through in-depth research in related fields such as molecular biology, immunology, and neurobiology, so as to yield more in-depth information for the ICI.
